# The first central precocious puberty proteomic profiles revealed multiple metabolic networks and novel key disease-associated proteins

**DOI:** 10.18632/aging.203676

**Published:** 2021-11-08

**Authors:** Chunlin Wang, Qingqing Chen, Ke Yuan, Minfei He, Jianfang Zhu, Yanlan Fang, Jianhong Hu, Qingfeng Yan

**Affiliations:** 1 Department of Pediatrics, The First Affiliated Hospital, College of Medicine, Zhejiang University, Hangzhou, Zhejiang Province, China; 2 Hailiang Hospital, Zhuji, Zhejiang Province, China; 3 College of Life Science, Zhejiang University, Hangzhou, Zhejiang Province, China

**Keywords:** central precocious puberty, proteome

## Abstract

Though central precocious puberty (CPP) as a disease that seriously affects the development of a child is increasing year by year, treatment options remain limited and is the same as the 1980s’ method. These are mainly due to the complex pathogenesis of central precocious puberty. Therefore, systems biology approach to identify and explore the multiple factors related to the pathogenesis of central precocious puberty is necessary. Our data established the first proteome profile of CPP revealed 163 down-regulated and 129 were up-regulated differentially expressed proteins. These altered proteins were primarily enriched in three metabolic process including energy metabolism, amino acid metabolism and nitrogenous base metabolism. The identified altered members of the metabolic signaling are valuable and potential novel therapeutic targets of central precocious puberty.

## INTRODUCTION

Central precocious puberty or named as GnRH-dependent precocious puberty (CPP) is diagnosed by premature physical and hormone characters of pubertal development usually in girls aged less than 8 years and in boys aged less than 9 years. CPP is always accompanied with growth acceleration and rapid bone maturation resulting to adult short stature [1]. CPP is mainly classified to three types. The most common type is named as idiopathic precocious puberty (ICPP) without central nervous system lesions. The second type is due to organic precocious puberty (intracranial lesions). The last type is transformed from peripheral precocious puberty [2]. Our study focused on exploring the molecular mechanism of the first type. The incidence of central precocious puberty increased significantly year by year and the age of onset is gradually earlier worldwide [3]. Unfortunately, the mechanism of central precocious puberty is still unclear due to the timing of pubertal onset is influenced by a complex interaction of genetic, epigenetic factors and environmental. Systematically to study the molecular mechanism of central precocious puberty is necessary for the diagnosis and treatment.

Currently, mutations in the kisspeptin system KISS1/KISS1R (peptide products of the kisspeptin-1/ peptide products of the kisspeptin-1 receptor), MKRN3 (makorin ring finger protein 3), and DLK1also named Pref-1 (Delta like non-canonical Notch ligand 1) have been identified as causal variants leading to CPP [4]. KISS1 and KISS1R are neurotransmitter and neurotransmitter receptor of GnRH respectively that directly control GnRH release [5]. MKRN3 functions as a ubiquitin E3 ligase to be essential for the puberty initiation [6]. Recent study showed that DLK1 is important for adipose tissue homeostasis and neurogenesis, and regulates Notch signaling to control the timing of puberty [7]. However, many areas remain to be explored such as KISS1/KISS1, MKRN3, and DLK1 targeting signals and the members, and other factors involved in central precocious puberty. Moreover, the timing of puberty is genetically determined and sensitive to numerous internal and external cues as metabolic signals [8]. And it was also reported central precocious puberty may be a manifestation of endocrine dysfunction in pediatric patients with mitochondrial disease [9]. However, which metabolic signals and metabolic members involving in central precocious puberty are unknown. Therefore, a systematic study is necessary to identify more members related to the cause of the disease and help to understand the complexity of the pathogenesis of central precocious puberty.

Proteomics study can identify and quantify diverse proteins involving complex biological processes [10]. To our best known, there are seldom researches about proteome with central precocious puberty. In this study, we used label-free technology combined with HPLC/LC-MS/MS analysis to identify the whole peripheral blood plasma proteome of central precocious puberty to identify the differentially expressed proteins (DEPs) and screen the candidate biomarkers. Our data was the first proteome profile of CPP and showed multiple metabolisms related differentially expressed proteins involved in central precocious puberty that maybe novel potential diagnostic and therapeutic targets.

## MATERIALS AND METHODS

### Central precocious puberty patients’ enrollment and the proteomic profile

**Figure 1:**
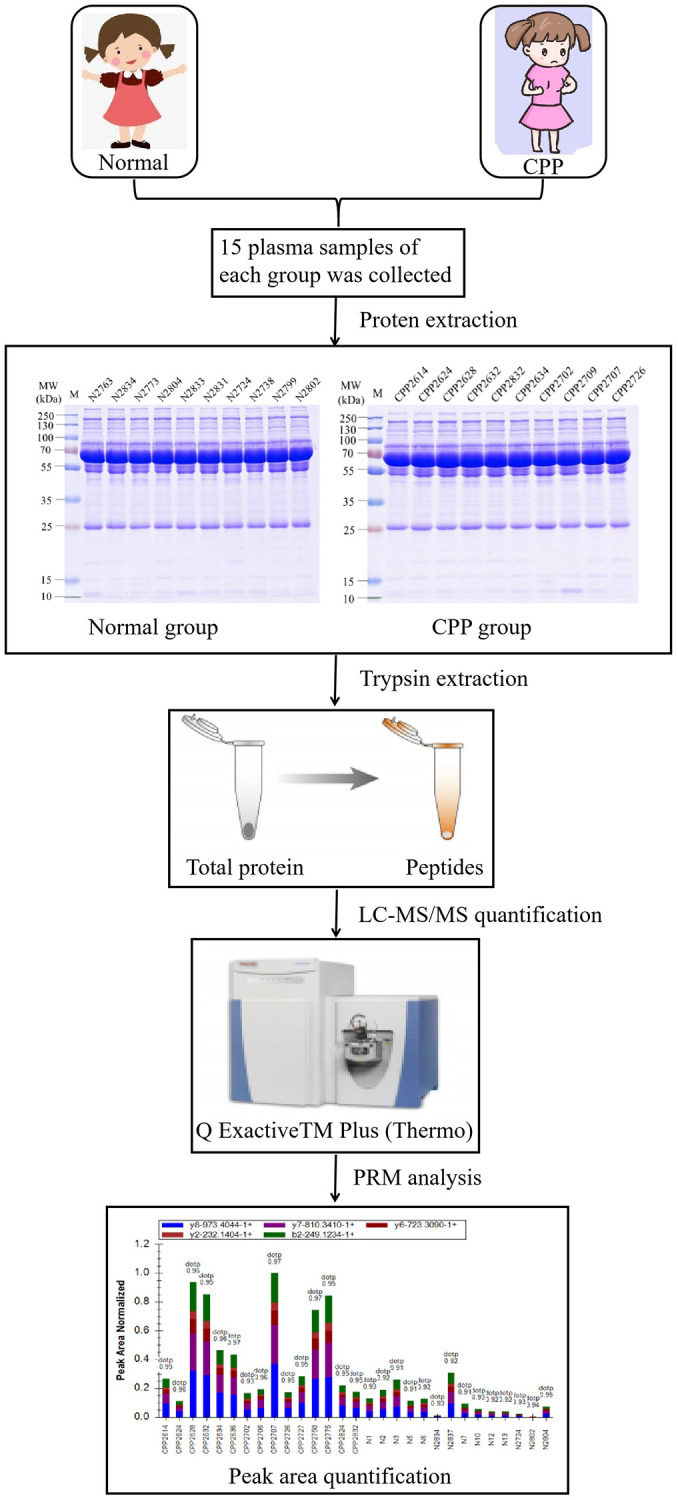
The workflow of proteomic analysis between normal and CPP group.

A total of 15 girls hospitalized with precocious puberty were enrolled from the Department of Pediatrics, the First Affiliated Hospital, College of Medicine, Zhejiang University in 2020. Central precocious puberty was confirmed according to the diagnostic criteria of CPP. The mainly inclusion criteria were as follows: 1. The chronological age was less than 8 years old and breast developed more than six months. 2. The bone age exceeded chronological age more than 1 year. 3. Ovarian volume greater than 1.0 ml. 4. The peak luteinizing hormone (LH_peak_) is more than 5 mIU/ml and LH_peak_/FSH_peak_ is more than 0.6 by LHRH test. 5. Organic central precocious puberty was excluded. Eventually, 15 girls diagnosed as central precocious puberty were qualified. Their mean age is 7.06 ± 1.37 years and bone age are 9.27 ± 0.31 years. BMI is 15.03 ± 1.27 kg/m^^2^. Luteinizing hormone (LH) at base line is 0.93 ± 0.33 mIU/ml and the peak LH is 18.48 ± 5.78 mIU/ml. Follicle-stimulating hormone (FSH) at base line is 3.14 ± 0.39 mIU/ml and the peak of FSH is 15.30 ± 1.25 mIU/ml. The LH_peak_/FSH_peak_ ratio is 1.17 ± 0.30. The left ovary volume is 2.45 ± 0.36 ml and the right is 2.22 ± 0.28 ml. 15 healthy girls were enrolled as normal group. Neither their parents nor their families had a history of precocious puberty. They were all from the Hailiang Primary School in Zhuji, Zhejiang Province, China. Their mean age was 7.19 ± 0.86 years and had no precocious puberty or puberty (Breast development, B1 phase) and BMI is 16.78 ± 1.48 kg/m^2^. The research protocol conformed to the ethical guidelines of the Declaration of Helsinki was approved by Clinical Research Ethics Committee of the First Affiliated Hospital, College of Medicine, Zhejiang University (No. 2020-300). To analyze the global protein expression atlas in central precocious puberty (CPP group) and healthy girls (Normal group), we collected the plasma and utilized LC-MS/MS to analyze the sample following the workflow described in Figure 1. As we known, our data firstly mapped the proteomic profile in central precocious puberty.

### Plasma samples collection

Blood samples from all participators were collected into plastic K_2_-EDTA tubes before breakfast after fasting for at least 8 hours. All samples were immediately manually inverted for 10 times and centrifuged at 12000 g at 4°C for 10 min. Finally, the supernatant was collected and the protein concentration was determined with BCA kit according to the manufacturer’s instructions of Pierce^™^ Top 12 Abundant Protein Depletion Spin Columns Kit (Thermo Scientific). Plasma samples were stored at −80°C until proteomic analysis.

### Trypsin digestion

For digestion, the protein solution was reduced with 5 mM dithiothreitol for 30 min at 56°C and alkylated with 11 mM iodoacetamide for 15 min at room temperature in darkness. The protein sample was then diluted by adding 100 mM TEAB to urea concentration less than 2 M. Finally, trypsin was added at 1:50 trypsin-to-protein mass ratio for the first digestion overnight and 1:100 trypsin-to-protein mass ratio for a second 4 h-digestion. The peptides were recovered by centrifugation at 12000 g for 10 min at room temperature, then the peptides were recovered with ultrapure water once, and the two peptide solutions were combined.

### LC-MS/MS analysis

The tryptic peptides were dissolved in 0.1% formic acid in 2% acetonitrile (solvent A), directly loaded onto a home-made reversed-phase analytical column (15-cm length,75 μm i.d.). The gradient was comprised of an increase from 4% to 20% solvent B (0.1% formic acid in 90% acetonitrile) over 96 min, 20% to 32% in 28 min, and climbing to 80% in 3 min then holding at 80% for the last 3 min, all at a constant flow rate of 500 nL/min on an EASY-nLC 1200 UPLC system. The peptides were subjected to NSI source followed by tandem mass spectrometry (MS/MS) in Exploris 480 (Thermo) coupled online to the UPLC. The electrospray voltage applied was 2.2 kv. The m/z scan range was 400 to 1200 for full scan, and intact peptides were detected in the Orbitrap at a resolution of 60 000. Peptides were then selected for MS/MS using NCE setting as 27 and the fragments were detected in the Orbitrap at a resolution of 30000. A data-dependent procedure that alternated between one MS scan followed by 20 MS/MS scans with 30.0s dynamic exclusion. Automatic gain control (AGC) was set at 7.5E4. Fixed first mass was set as 100 m/z.

### Database search

The resulting MS/MS data were processed using PD 2.4. Tandem mass spectra were searched against human uniport database concatenated with reverse decoy database. Trypsin/P was specified as cleavage enzyme allowing up to 2 missing cleavages. The mass tolerance for precursor ions was set as 10 ppm in First search and 5 ppm in Main search, and the mass tolerance for fragment ions was set as 0.02 Da. Carbamidomethyl on Cys was specified as fixed modification and acetylation modification and oxidation on Met were specified as variable modification. FDR was adjusted to <1% and minimum score for modified peptides was set >40.

### Bioinformatics methods

The Gene Ontology (GO) annotation proteome analysis was derived from the UniProt-GOA database (http://www.ebi.ac.uk/GOA) and the InterProScan soft. Proteins were classified by GO based on three categories: biological process (BP), cellular component (CC) and molecular function (MF). The Kyoto Encyclopedia of Genes and Genomes (KEGG) database was used to annotate protein pathway (metabolism, genetic information processing, cellular processes, rat disease, environmental information processing, drug development (http://www.genome.jp/kegg/). We used wolfpsort as a subcelluar localization predication soft to predict subcelluar localization. Wolfpsort is an updated version of PSORT/PSORTII for the prediction of eukaryotic sequences. GO annotation and enriched pathways with a corrected *P*-value <0.05 were considered statistically significant. All differentially expressed proteins database accessions or sequences were searched against the STRING database (version 10.5) for protein-protein interactions (PPI). Only interactions between the proteins belonging to the searched data set were selected, thereby excluding external candidates. STRING defines a metric known as “confidence score” to define interaction confidence; we used all interactions with a confidence score ≥0.7 (high confidence).

### Parallel reaction monitoring analysis

The selected proteins’ peptide samples were prepared for Parallel Reaction Monitoring (PRM) using the results of proteome analysis described above. The tryptic peptides were dissolved in 0.1% formic acid (solvent A) and directly loaded onto a home-made reversed-phase analytical column. Solvent B consisted of 0.1% formic acid in 98% acetonitrile and increased from 6% to 23% for 38 min, 23% to 35% for 14 min, 80% for 4 min, and was then held at 80% for an additional 4 min. The flow rate was a constant 700 nL/min on an EASY-nLC 1000 UPLC system. The peptides were subjected to the NSI source followed by tandem mass spectrometry (MS/MS) in Q Exactive^™^ Plus (Thermo) system that was coupled online to the UPLC. The electrospray voltage was 2.0 kV, the m/z scan range was 350 to 1000 (full scan), and intact peptides were detected in the Orbitrap at a resolution of 35,000. Peptides were then selected for MS/MS using a normalized collision energy (NCE) of 27, and the fragments were detected in the Orbitrap at a resolution of 17,500. A data-independent procedure alternated between one MS scan followed by 20 MS/MS scans. The AGC was 3E6 for full MS and 1E5 for MS/MS. The maximum IT was 20 ms for full MS and automatic for MS/MS. The isolation window for MS/MS was 2.0 m/z. The resulting MS data were processed using Skyline (ver. 3.6). For peptide settings, the enzyme was Trypsin [KR/P], and the maximum missed cleavage set was as 2. The peptide length was 8 to 25, variable modification was carbamidomethyl on Cys and oxidation on Met, and max variable modifications were 3. For transition settings, the precursor charges were 2 and 3, the ion charges 1 and 2, and the ion types were b, y, and p. The product ions were from ion 3 to last ion, the ion match tolerance was 0.02 Da.

### Data availability

All authors declare that all data are fully available without restriction.

## RESULTS

### Quality control of mass spectral data and total identified differentially expressed proteins in the proteomic profile

**Figure 2:**
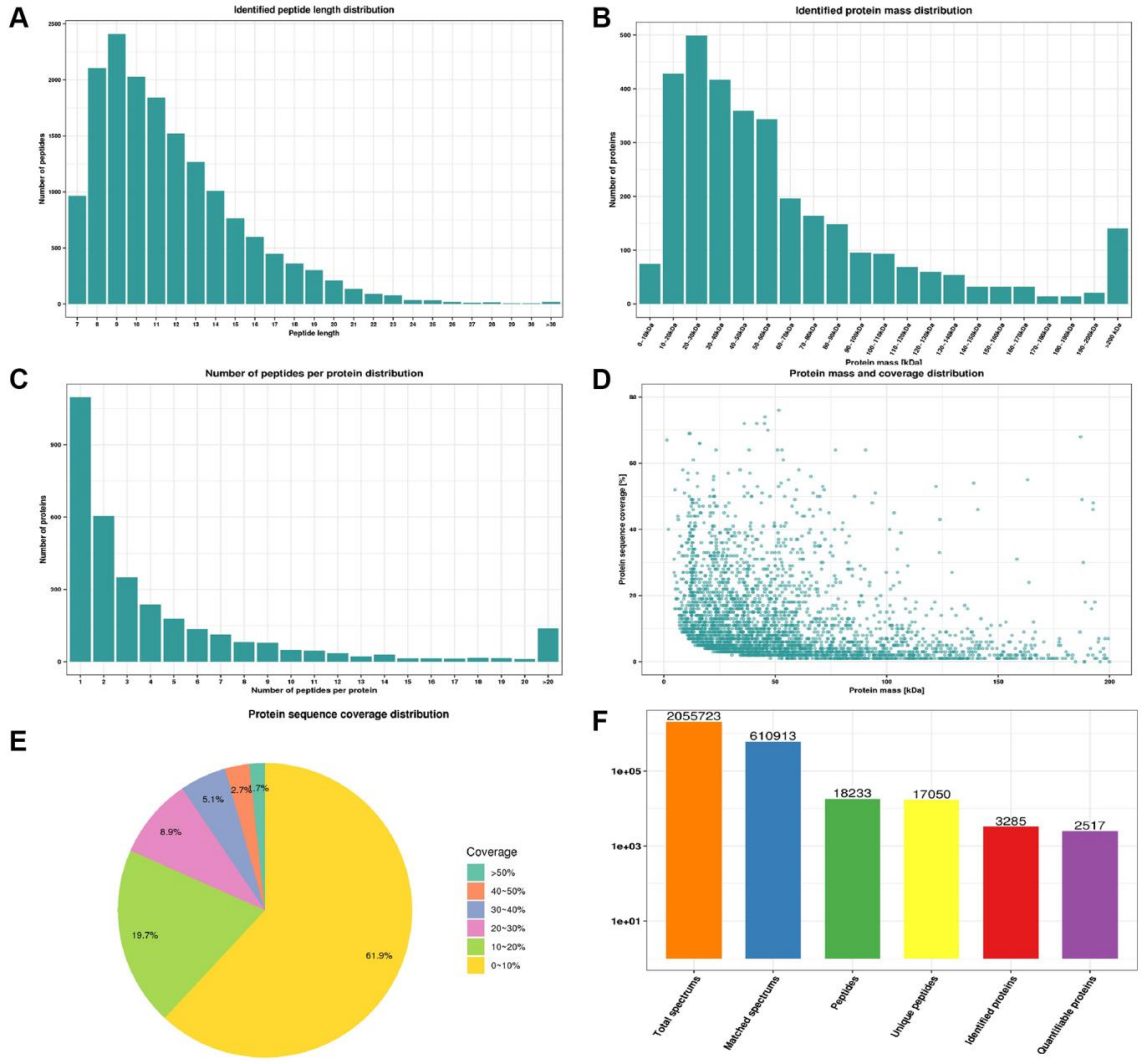
**Quality control indexes and proteome landscape between normal and CPP group.** (**A**) Identified peptide length distribution. (**B**) Identified protein mass distribution. (**C**) Number of peptides per protein distribution. (**D**) Protein mass and coverage distribution. (**E**) Protein sequence coverage distribution. (**F**) The number of total spectrums, matched spectrums, identified peptides, unique peptides, identified proteins and quantifiable proteins.

We firstly based on multidimensional quality control indexes including peptide length distribution, identified protein mass distribution, number of peptides per protein distribution, protein mass and coverage distribution and protein sequence coverage distribution to assess mass spectrometry-based proteomics data. Most peptides length distributed between 7–20 without less than 5 peptides (Figure 2A). Proteins above 10 kDa were distributed uniformly without obvious molecular weight shift (Figure 2B). Most proteins corresponded to more than two peptides with high precision and credibility (Figure 2C). The molecular weight of the protein was negatively correlated with the coverage (Figure 2D) and most proteins have a coverage of less than 20% (Figure 2E). In summary, our study identified 2055723 spectrums, 610913 matched spectrums, 18233 peptides, 17050 unique peptides, 3285 proteins and quantified 2517 proteins of which 163 were down-regulated and 129 were up-regulated in the CPP group compared to the Normal group (Figure 2F). The criterion for significant differences between two groups was ≥1.5-fold change and *p*-value < 0.05.

### Validation of quantitative proteomics results via parallel reaction monitoring

**Figure 3:**
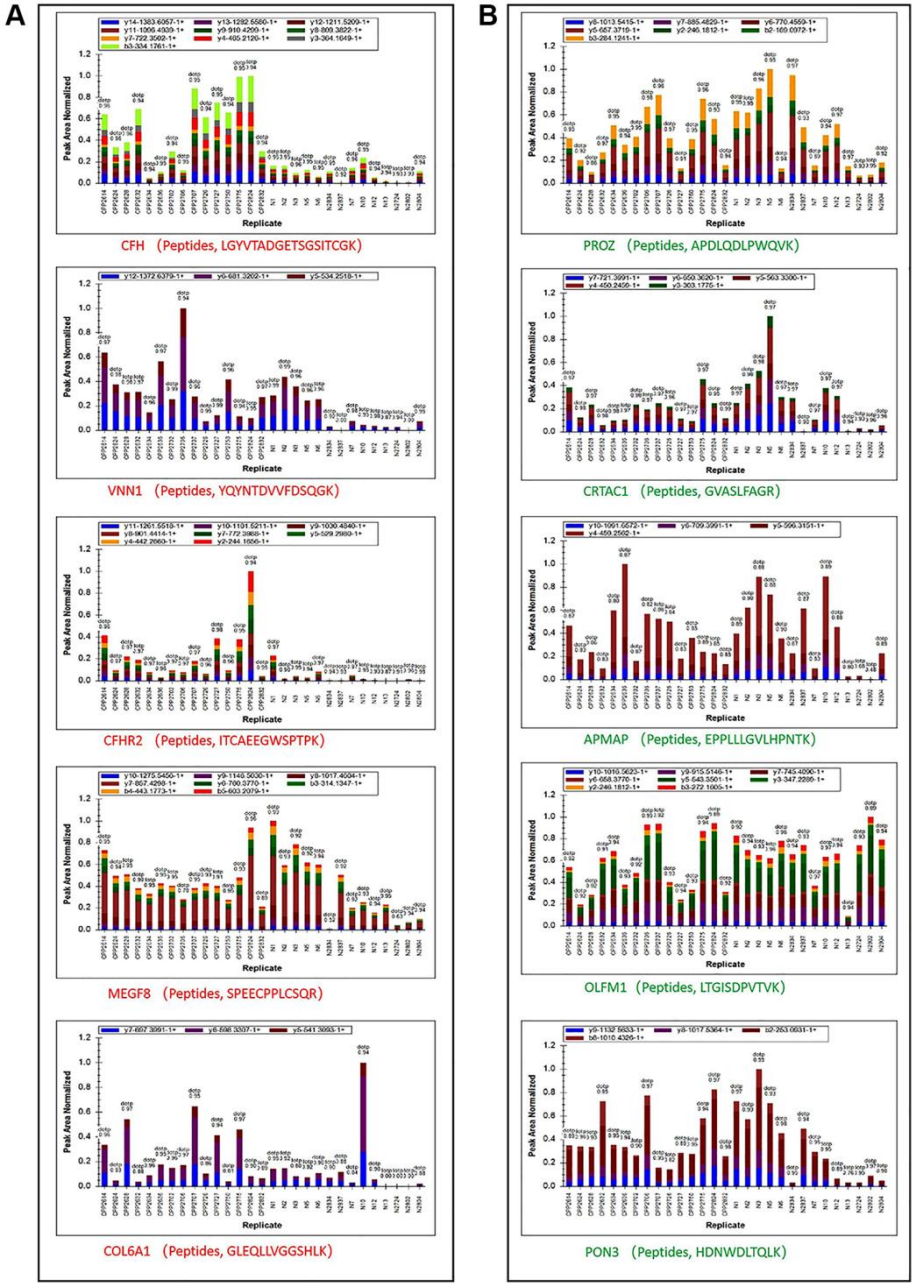
**Peptide ion peak area distribution of PRM.** (**A**) Ion peak area distribution of top 5 up regulated proteins’ peptides. (**B**) Ion peak area distribution of top 5 down regulated proteins’ peptides.

We then performed Parallel Reaction Monitoring (PRM) analysis to validate the differentially expressed proteins (DEPs) identified by quantitative proteomics. We selected the top five up-regulated and down-regulated, and 10 randomly selected DEPs (Protein accession: O43866, Q5SRP5, P19652, P02751, P02452, P22692, P00738, Q15848, P00918 and A0A0C4DH42) to analysis, and successfully quantitated and confirmed all up-regulated and down-regulated and other 9 randomly selected DEPs (Figure 3A and 3B, Table 1). The PRM and proteomic quantification results were highly consistent, and the PRM results had good correlation with the proteomics results that suggested the high quality and credibility of this first proteomic profile of central precocious puberty.

**Table 1:** Quantitative confirmation of top 5 up or down regulated and 10 randomly selected proteins.

**Protein Accession**	**Protein Name**	**CPP/N Ratio**	**CPP/N *P*-value**
**P08603**	**CFH**	**6.80**	**6.39E-07**
**O95497**	**VNN1**	**2.72**	**2.77E-03**
**V9GYE7**	**CFHR2**	**6.85**	**8.26E-06**
**Q7Z7M0**	**MEGF8**	**1.23**	**3.76E-02**
**A0A087X0S5**	**COL6A1**	**1.69**	**2.50E-01**
*P22891*	*PROZ*	*1.03*	*4.77E-01*
*A0A0C4DFP6*	*CRTAC1*	*0.86*	*4.70E-01*
*H0Y512*	*APMAP*	*0.73*	*8.08E-01*
*Q99784*	*OLFM1*	*0.82*	*3.07E-01*
*Q15166*	*PON3*	*1.17*	*8.39E-02*
***O43866***	***CD5L***	***0.87***	***7.49E-01***
***Q5SRP5***	***APOM***	***0.95***	***9.52E-01***
***P19652***	***ORM2***	***0.69***	***8.14E-01***
***P02751***	***FN1***	***3.87***	***1.16E-04***
***P02452***	***COL1A1***	***1.92***	***2.90E-04***
***P22692***	***IGFBP4***	***1.18***	***1.95E-01***
***P00738***	***HP***	***0.46***	***8.92E-01***
***Q15848***	***ADIPOQ***	***0.27***	***1.00E-02***
***P00918***	***CA2***	***1.92***	***5.04E-03***

The bold and the italic texts are the top 5 up or down regulated proteins respectively. The bold italic texts are randomly selected differentially expressed proteins.

### GO function, subcellular localization and COG/KOG categories analysis of differentially expressed proteins

**Figure 4:**
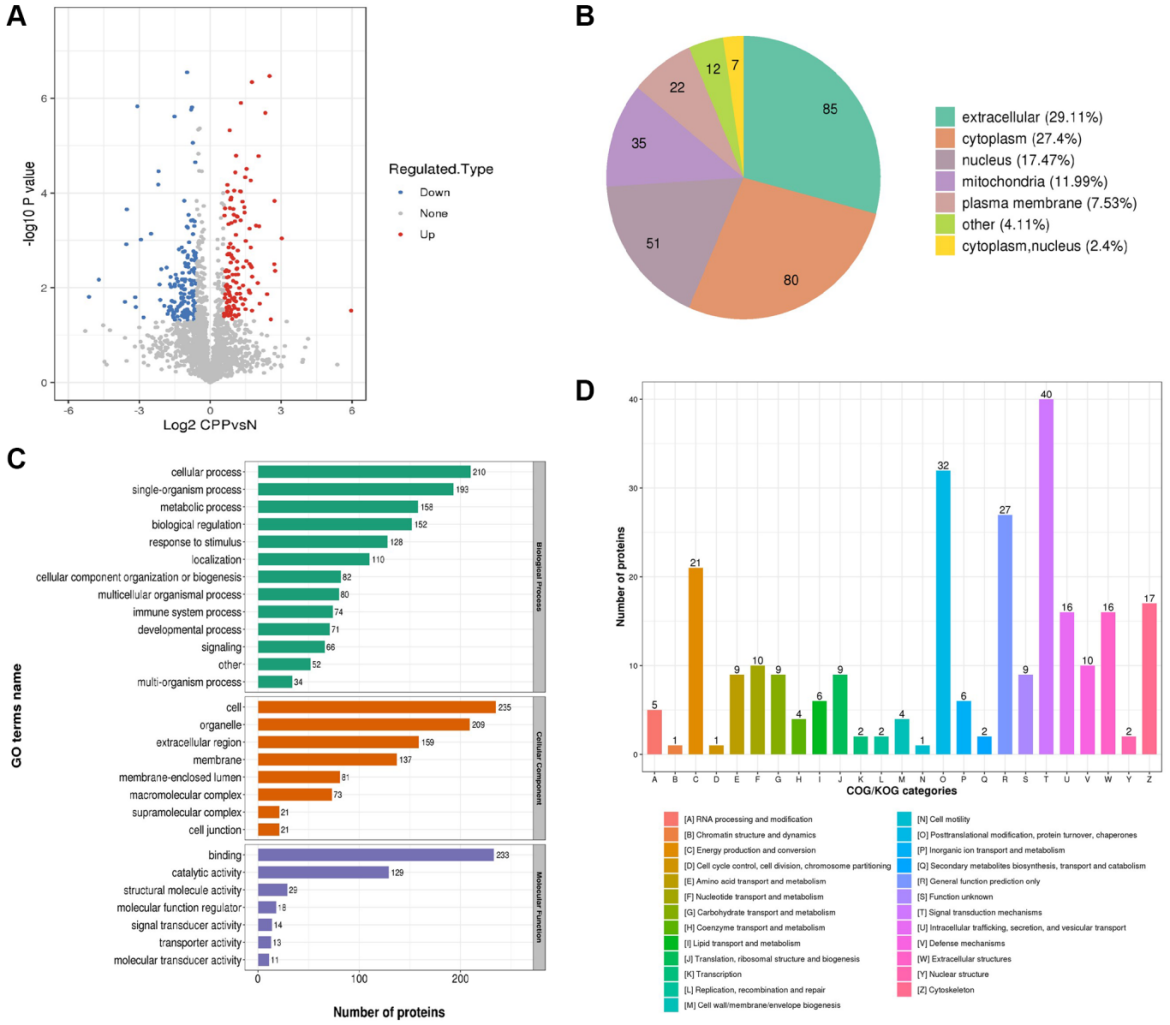
**Global proteomics analyses of the identified differentially expressed proteins in CPP.** (**A**) The horizontal axis and vertical axis of the volcano plot was the logarithmically converted value of the relative quantitative value of the protein and logarithmically converted *p*-value after the log-log conversion, respectively. In the volcano plot, the red dot indicated significantly up regulated proteins and the blue dot indicated significantly down regulated proteins. The *p*-value was calculated using the two-sample *t*-test method. (**B**) Clusters of Orthologous Groups of differentially expressed proteins. (**C**) Sub cellular Localization and classification of differentially expressed proteins. (**D**) The up regulated proteins and down regulated proteins between two groups via Enrichment of GO analysis.

The overall of all 292 differentially expressed proteins was visualized via a volcano plot as shown in Figure 4A. Then the subcellular localization of 292 differentially expressed proteins (129 up-regulated and 163 down-regulated) were predicted and analyzed. The top three subcellular localization were extracellular (29.11%), cytoplasm (27.4) and nucleus (17.47%) as shown in Figure 4B. Interestingly, 11.99% DEPs were in mitochondria (Figure 4B) and previous study showed central precocious puberty may be a manifestation of endocrine dysfunction in pediatric patients with mitochondrial disease as we mentioned before [9]. Next, we classified all DEPs into biological process (BP), cellular component (CC) and molecular function (MF) based on three biological functions. The top three of BP were cellular process (210 DEPs), single-organism process (193 DEPs) and metabolic process (153 DEPs) (Figure 4C, green part). The top three of CC were cell (235 DEPs), organelle (209 DEPs) and extracellular region (159 DEPs) (Figure 4C, red part). This is consistent with the subcellular localization analysis showed 29.11% DEPs were located extracellular (Figure 4B). The top three of MF were binding (233 DEPs), catalytic activity (129 DEPs) and structural molecule activity (29 DEPs) (Figure 4C, purple part). Finally, DEPs were categorized based on homology and functionality. The top category was signal transduction mechanisms including 40 DEPs and consistent with the GO annotation showed 193 DEPs participating in single-organism process (Figure 4C). The “O” category (posttranslational modification, protein turnover, chaperones), the “R” category (general function prediction only) and the “C” category (energy production and conversion) clustered major DEPs and were 32, 27 and 21 respectively (Figure 4D). Interestingly, the data of Figure 4B–4D all revealed metabolic process especially energy metabolism maybe closely related to central precocious puberty.

### PPI network of differentially expressed proteins in central precocious puberty

**Figure 5:**
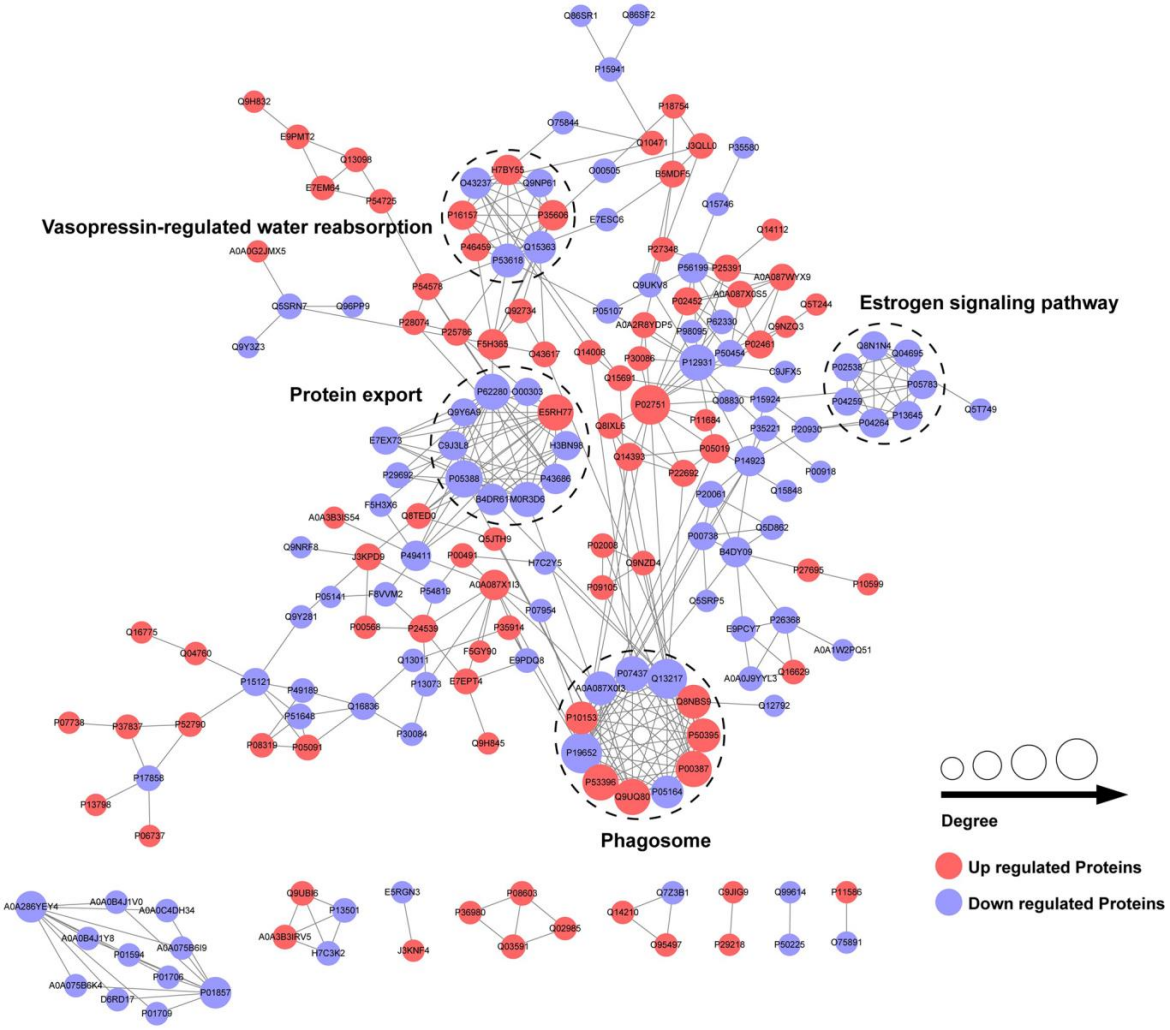
PPI network of differentially expressed proteins in CPP.

To explore the interactions among the differentially expressed proteins between the CPP group and the Normal group, we conducted a PPI network by searching the PPI database (https://string-db.org). Then, we performed a clustering analysis to find highly interconnected clusters based on the PPI network. As shown in Figure 5, a total of 4 clusters (non-clustered proteins are not shown) were detected including vasopressin-regulated water reabsorption, protein export, estrogen signaling pathway and phagosome. Notably, most DEPs of protein export cluster and all DEPs of estrogen signaling pathway cluster were down-regulated that suggests a potential negative regulation in central precocious puberty.

### Protein domain and KEGG pathway enrichment and heatmap analysis of differentially expressed proteins in central precocious puberty

**Figure 6:**
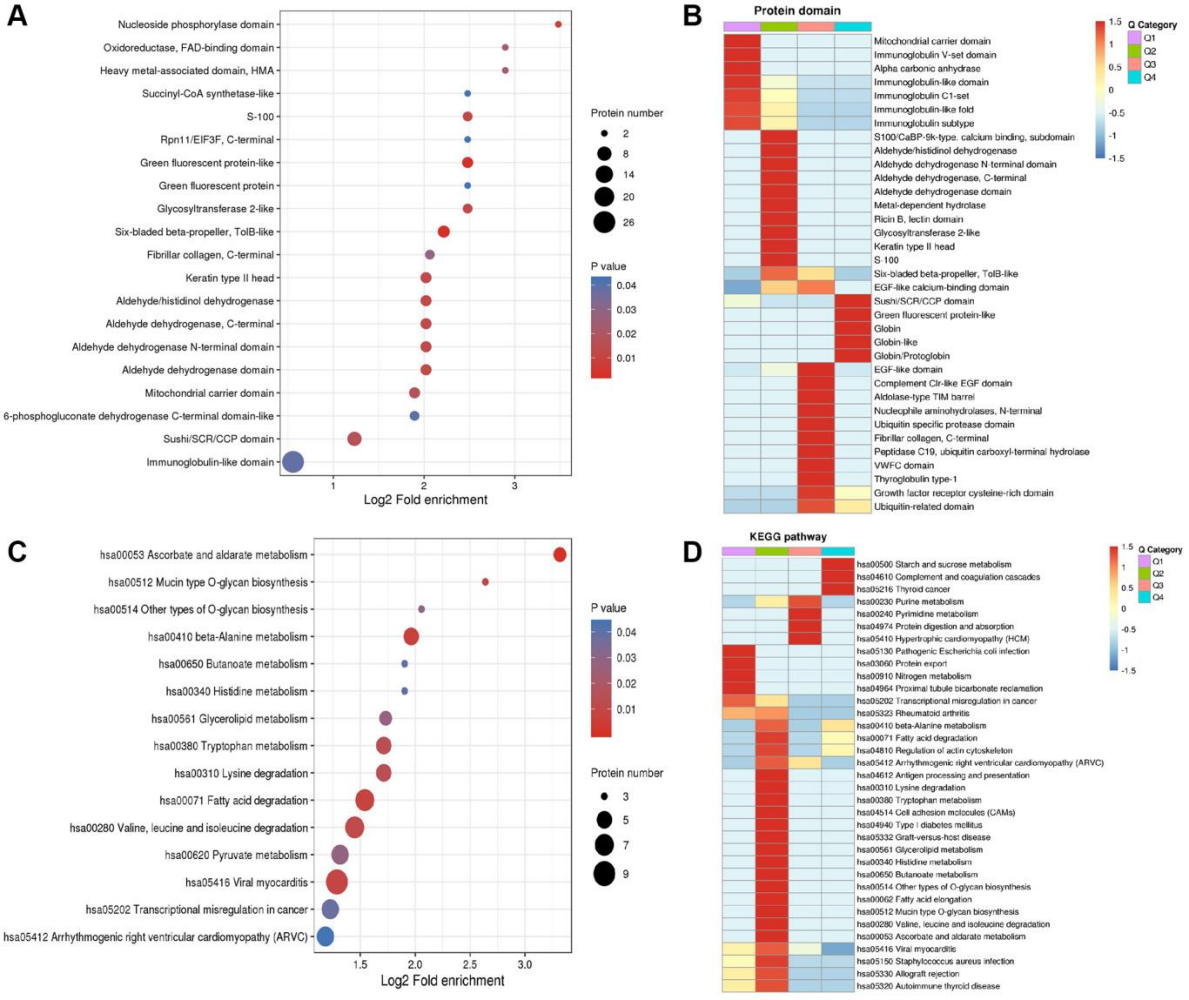
**Enrichment analysis and heatmap analysis of protein domains and KEGG pathway.** (**A**) Enrichment analysis of protein domains. (**B**) Heatmap analysis of proteins domains. (**C**) Enrichment analysis of KEGG pathway. (**D**) Heatmap analysis of KEGG pathway.

DEPs were first enriched (Figure 6A and 6C) and then classified to four subgroups named as Q1–4. The four subgroups were hierarchically clustered according to related functions via heatmap. DEPs with mitochondrial carrier domain were significant up-regulated in Q1 category (Figure 6B). A total of 35 pathways were generated from the KEGG pathway analysis based on the differentially expressed proteins between the CPP group and the Normal group.

Most significant regulated KEGG pathways are related to metabolism including starch and sucrose metabolism (hsa00500 including P52790 and P06737) in Q4, purine metabolism (hsa00230 including P00568, J3KPD9 and P00491) in Q3, pyrimidine metabolism (hsa00240 including J3KPD9 and P00491) in Q3, nitrogen metabolism (hsa00910 including P00918 and P22748) in Q1, beta-Alanine metabolism (hsa00410) in Q2 (including P30084, P51648 and P49189) and Q4 (including O95822 and P05091), tryptophan metabolism (hsa00380 including Q16836, P30084, P51648 and P49189) in Q2, glycerolipid metabolism (has00561 including P51648 and P49189) in Q2, histidine metabolism (hsa00340 including P51648 and P49189) in Q2, butanoate metabolism (hsa00650 including Q16836 and P30084) in Q2, ascorbate and aldarate metabolism (hsa00053 including P51648 and P49189) in Q2 (Figure 6D).

## DISCUSSION

Central precocious puberty estimated the incidence at 0.2% of girls or 1.1 per 100,000 girls and the incidence is increasing year by year [11, 12]. Though knowledge regarding central precocious puberty continued to enrich, the exact control signal and members of central precocious puberty remains a mystery. It is known that endogenous signal plays a primary role combined with complex environmental and nutritional factors to affect the occurrence and development of central precocious puberty [13, 14]. However, until now only the kisspeptin system KISS1/KISS1R, MKRN3, and DLK1 or Pref-1 identified in sporadic or familial central precocious puberty cases have been confirmed causal variants leading to CPP [4]. In clinical management, long-acting hypothalamic gonadotropin-releasing hormone (GnRH) agonists have been widely used as the gold-standard treatment but with several adverse effects including the desensitization of luteinizing hormone release, the regression of puberty and the delay progress of bone age [15, 16]. Short-acting GnRH or substitutive therapy of GnRH agonists urgently needs to establish. However, the molecular mechanism of central precocious puberty is unclear and the exact therapeutic targets are scarce. Since the mid-1980s, long-acting GnRH agonists are almost the only available treatment and child patients are usually required to take the drugs for more than two years [17]. Moreover, the laboratory experimental markers for the diagnosis of central precocious puberty remain absent.

Our present study first identified 292 differentially expressed proteins including 163 down-regulated and 129 up-regulated DEPs in the CPP group compared to the Normal group (Figure 2F). Additionally, KEGG pathway analysis revealed several metabolic processes be closely related to central precocious puberty (Figure 6). Especially, our data further implied energy metabolic signal may play a vital role in central precocious puberty supported by numerous evidences including 11.99% mitochondria subcellular location of DEPs (Figure 4B), 21 DEPs enriched in “energy production and conversion” (Figure 4D) and DEPs with mitochondrial carrier domain were significant up-regulated (Figure 6B).

Clinical reports suggested central precocious puberty was closely connected with alterations in metabolic factors [18–20]. Central precocious puberty patients with DLK1 defection accompanied with metabolic disturbance including hyperlipidemia, obesity, glucose intolerance et al. [18, 19]. Adverse metabolic abnormality persistently existed during the therapeutic process even after GnRHa treatment [20]. Consistent with these reports, our data confirmed metabolic regulation as the major changes in central precocious puberty and quantificationally identified related metabolic regulation members in detail. In summary, energy metabolism (hsa00500 starch and sucrose metabolism), amino acid metabolism (hsa00410 beta-Alanine metabolism, hsa00380 tryptophan metabolism and hsa00340 histidine metabolism) and nitrogenous base metabolism (hsa00230 purine metabolism and hsa00240 pyrimidine metabolism) are three major related metabolic regulation in central precocious puberty.

**Figure 7:**
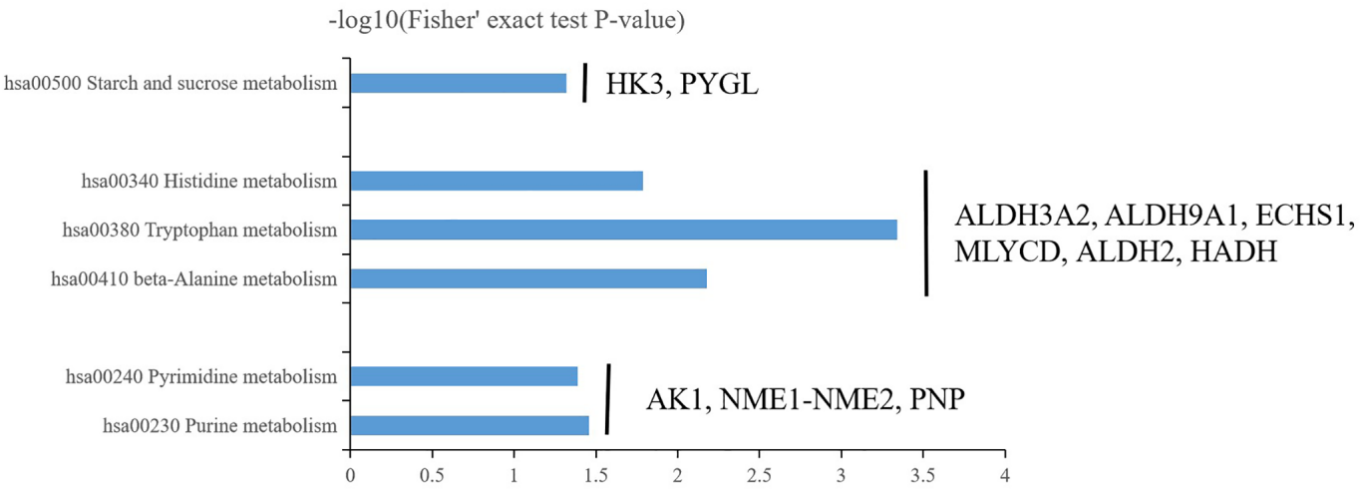
Three major enrichment metabolisms in central precocious puberty including energy metabolism (hsa00500 starch and sucrose metabolism), amino acid metabolism (hsa00410 beta-Alanine metabolism, hsa00380 tryptophan metabolism and hsa00340 histidine metabolism) and nitrogenous base metabolism (hsa00230 purine metabolism and hsa00240 pyrimidine metabolism).

In energy metabolism (hsa00500 starch and sucrose metabolism), Hexokinase-3 (HK3, P52790) and glycogen phosphorylase liver isoform (PYGL, P06737) was up-regulated 2.825233927 and 2.459379777-fold respectively in central precocious puberty (Figure 7). HK3 belongs to the hexokinase family and is a glycolytic enzyme phosphorylating glucose to glucose-6-phosphate [21, 22]. The hexokinase family contains four hexokinase isozymes HK1-4 that sense glucose level and the sequence of HK1 to HK3 are highly homology but exhibit unique tissue distribution that results to distinct physiologic function [23, 24]. HK3 is found with a high expression in uterus, placenta and adipose [21]. In particular cells (granulocytes) HK3 contributes major hexokinse activity (~70–80%) [25]. Our data only identified HK3 without HK1, 2, 4 that suggested HK3 may also play a particular role in central precocious puberty and further explore the distribution of HK3 in central precocious puberty patients’ tissue would be important to clarify its functions. PYGL is the member of glycogen phosphorylase (PYG) family including three different tissue distribution members PYGM (the muscle isoform), PYGL (the liver isoform) and PYGB (the brain isoform) that are the key rate-limiting enzyme in glycogenolysis [26, 27]. Our data only identified the significant up-regulated of PYGL that implied the special liver glycogen homeostasis closely related to central precocious puberty (Figure 7). Another identified differentially expressed protein PFKL (P17858, ATP-dependent 6-phosphofructokinase, liver type) further confirmed the speculate. There are two 6-phosphofructokinase isomers PFKM (the muscle isoform) and PFKL (the liver isoform) in humans and only the liver isoform PFKL was identified [28]. In conclusion, central precocious puberty affects liver glycogen homeostasis not muscle or brain. Furthermore, PHKA1 (phosphorylase kinase alpha 1) and PYGL have been reported as two key regulators during glycogen metabolism to balance the supplement of the glucose but the expression of PHKA1 was not changed in central precocious puberty that suggested the regulation of glycogen metabolism only via PYGL not PHKA1.

In acid metabolism (hsa00410 beta-Alanine metabolism, hsa00380 tryptophan metabolism and hsa00340 histidine metabolism), Malonyl-CoA decarboxylase mitochondrial (MLYCD, O95822) and Aldehyde dehydrogenase mitochondrial (ALDH2, P05091) was up-regulated 2.200942237 and 2.745247991-fold respectively in central precocious puberty (Figure 7). Enoyl-CoA hydratase mitochondrial (ECHS1, P30084), Aldehyde dehydrogenase family 3 member A2 (ALDH3A2, P51648), 4-trimethylaminobutyraldehyde dehydrogenase (ALDH9A1, P49189) and Hydroxyacyl-coenzyme A dehydrogenase mitochondrial (HADH, Q16836) was down-regulated 0.632687067, 0.638665838, 0.627453085 and 0.582303316-fold respectively in central precocious puberty (Figure 7). Interestingly, most DEPs (MLYCD, ALDH2, ECHS1 and HADH) involved in acid metabolism was predicted to locate in mitochondria or contained mitochondrial carrier domain (Figure 4B, Figure 6B) that suggested central precocious puberty may affect mitochondria related acid metabolism. There are few reports about the role of MLYCD in beta-Alanine metabolism that is known to accelerate oxidation via the peroxisome to inhibit the activity of pyruvate dehydrogenase [29]. ALDH2 can transform β-aminopropion-aldehyde to β-alanine [30] and is widely studied as an important cardioprotective factor [31]. Activate ALDH2 promote mitochondrial homeostasis and suppress mitochondrial ROS (reactive oxygen species) [32, 33]. In our results, both MLYCD and ALDH2 was obviously up-regulated that MLYCD may a marker of ROS in central precocious puberty and ALDH2 may play an endogenous protective role. *ECHS1* mutation or ECHS1 deficiency is known as a primary cause leading to lactic acidosis in neonate that maybe related to mitochondrial fatty acid oxidation disorder [34, 35]. Our data showed the down-regulation of ECHS1 that suggested the identification of *ECHS1* mutation and the detection of lactic acid changes maybe valuable in specific central precocious puberty patients. HADH is reported as a common cause of hyperinsulinemia and the function of HADH needs further to determine in central precocious puberty [36]. Furthermore, the function of ALDH3A2 and ALDH9A1 should be pay special attention because ALDH3A2 and ALDH9A1 were differentially expressed in all three acid metabolisms (Figure 7). However, the existing studies on ALDH3A2 and ALDH9A1 are less. *ALDH3A2* mutation has been reported to cause Sjögren-Larsson syndrome [37, 38] and the variation of ALDH9A1 maybe a related pathogenic factor of renal cancer [39]. Further to explore the role of ALDH3A2 and ALDH9A1 in central precocious puberty will be interesting.

In nitrogenous base metabolism (hsa00230 purine metabolism and hsa00240 pyrimidine metabolism), Adenylate kinase isoenzyme 1 (AK1, P00568), NME1-NME2 read through (NME1-NME2, J3KPD9) and Purine nucleoside phosphorylase (PNP, P00491) were all up-regulated 1.582035928, 1.637353964 and 1.975223898-fold respectively (Figure 7). AK1 is selected as a prognostic indicator in acute myeloid leukemia patients treated with chemotherapy [40]. NME1 play a potential role to regulated neuroblastoma or hepatocellular carcinoma pathogenesis and NME2 can inhibit gastric cancer metastasis [41–43]. PNP play an important in ATP binding and the stabilization of RNaseII [44, 45]. The function of AK1, NME1-NME2 and PNP in central precocious puberty needs further research to clarify the relationship between nitrogenous base metabolism and CPP pathogenesis.

## CONCLUSIONS

Overall, our data provided a valuable profile with high quality and reliability that confirmed via multiple quality control indexes and PRM. The results suggested that multiple metabolic regulation may participate in central precocious puberty. In addition, these data further provided candidate differentially expressed proteins that may be novel potential targets for the future research of central precocious puberty diagnoses and treatment.
